# Seroprevalence of antibodies to measles, mumps, and rubella, and serologic responses after vaccination among human immunodeficiency virus (HIV)-1 infected adults in Northern Thailand

**DOI:** 10.1186/s12879-016-1499-x

**Published:** 2016-04-30

**Authors:** Romanee Chaiwarith, Jutarat Praparattanapan, Khanuengnit Nuket, Wilai Kotarathitithum, Khuanchai Supparatpinyo

**Affiliations:** Division of Infectious Diseases, Department of Medicine, Faculty of Medicine, Chiang Mai University, Chiang Mai, 50200 Thailand; Research Institute for Health Sciences, Chiang Mai University, Chiang Mai, 50200 Thailand

**Keywords:** MMR, Vaccination, HIV, Serologic response

## Abstract

**Background:**

After the global implementation of national immunization programs for prevention of measles, mumps, and rubella (MMR), the prevalences of protective antibodies to these viruses are high in general population. However, there are limited data among human immunodeficiency virus (HIV)-1 infected individuals. This study aimed to determine the seroprevalence of antibodies to these viruses, and the serologic responses after vaccination among HIV-infected adults in Northern Thailand.

**Methods:**

A cross-sectional study was conducted in 500 HIV-infected adults, aged 20–59 years, receiving combination antiretroviral therapy, CD4 cell count ≥200 cells/mm^3^, and plasma HIV-1 RNA <50 copies/mL, and 132 HIV-uninfected controls, aged 20–59 years, at Chiang Mai University Hospital during July and August 2011. Prevalences of protective antibodies to these viruses as well as serologic responses after MMR vaccination in those without protective antibody to at least one of the three viruses were compared between groups.

**Results:**

The prevalences of protective antibodies to measles, mumps, and rubella were 94.2, 55.0, and 84.6 % among HIV-infected adults, and 97.7, 67.5, and 89.4 % among HIV-uninfected controls, respectively. The prevalence of protective antibody to mumps was significantly lower in HIV-infected adults (*p*-value = 0.010). MMR vaccination was done in 249 HIV-infected and 46 HIV-uninfected controls; at week 8 to 12 after vaccination, the seroprotective rates against measles, mumps, and rubella in HIV-infected adults were 96.4, 70.7, and 98.0 %, respectively, whereas those in HIV-uninfected controls were 100, 87, and 100 %, respectively. No serious adverse effects were observed.

**Conclusions:**

In contrast to measles and rubella, the prevalence of protective antibody to mumps was low in both HIV-infected adults and HIV-uninfected controls in northern Thailand. The seroprotective rates after MMR vaccination in both groups were considerably high, except only for mumps. Therefore, MMR vaccination should be considered in all HIV-infected adults receiving antiretroviral therapy with undetectable plasma HIV-1 RNA and CD4 cell count ≥200 cells/mm^3^.

**Trial registration:**

ClinicalTrials.gov: NCT02724852, registered on March 31, 2016.

## Background

Measles, mumps, and rubella have long been regarded as diseases of childhood [1–3]. However, when infections from these viruses occur in adults, they are often more severe [1–3]. The serious complications may occur particularly in pregnant women [2]. The incidence of measles, mumps, and rubella has decreased dramatically in countries where measles, mumps, and rubella (MMR) vaccination is integrated into the national immunization program, including Thailand [4–6]. The first dose of measles vaccination in children aged 9–12 months and the second dose in first grade students were incorporated into the Thai national immunization program in 1984 and 1996, respectively. Rubella vaccination in first grade students was incorporated into the program 1993. The vaccine against single virus was replaced by MMR vaccine in 1997 [7]. The Thai national survey in 2003 and 2004 showed that the coverages of the first dose of measles vaccine among children aged 9–12 months and MMR vaccine among the first grade students were 96 and 94 %, respectively [8, 9]. In general, measles vaccination produces protective antibody in 88–95 % of children [10, 11]. The seroprevalence study among Thai population in 2004 revealed that 81 % (95 % CI 78.8–83.5) of 1092 serum specimens tested, 89 % (95 % CI 86.8–91.0) of 899 specimens tested, and 82 % (95 % CI 78.9–84.0) of 911 specimens tested had protective antibody to measles, mumps, and rubella, respectively [6]. Although the vaccine coverage and the rate of protective antibody to these viruses are quite high in Thai population, periodic outbreaks have been reported and might be attributed to the waning of antibody after a long period of time [12]. 

A study in Thai HIV-infected children receiving combination antiretroviral therapy (cART) reported that the prevalence of protective antibody to measles was only 42 % [13]. A study among HIV-infected adults in the United States reported that the prevalences of protective antibodies to measles, mumps, and rubella were 67, 91, and 95 %, respectively [14]. MMR revaccination has been studied in Thai children aged over 5 years and had CD4 > 15 % for at least 3 months after receiving cART. The study showed that the seroprotective rates against measles, mumps, and rubella declined overtime after vaccination [15]. In adults, a study in Mexico showed the early loss of measles antibody after 24 months of vaccination despite receiving cART [16]. These evidence suggested that loss of antibody responses may occur in HIV-infected people after vaccination, particularly in advanced diseases.

Up to date, there have been limited data regarding the prevalences of protective antibodies to measles, mumps, and rubella among Thai HIV-infected adults. We, therefore, conducted this study to determine 1) the prevalences of protective antibodies to measles, mumps, and rubella in HIV-infected adults and HIV-uninfected controls in northern Thailand, 2) the serologic responses at 8 to 12 weeks, and 48 weeks after MMR vaccination in those without protective antibody to at least one of the three viruses, and 3) adverse effects of MMR vaccination.

## Methods

### Study design, and population

The first phase of the study was a cross-sectional study to determine the prevalences of MMR-specific antibodies in HIV-infected adults and HIV-uninfected controls. HIV-infected adults receiving care at the HIV clinic of the Chiang Mai University Hospital between July and August 2011 and met the following criteria were consecutively enrolled: 1) 20–59 years old, 2) receiving cART, 3) CD4 cell count ≥200 cell/mm^3^ within 6 months before enrolment, 4) plasma HIV-1 RNA <50 copies/mL, and 5) ability to provide written informed consent. HIV-uninfected controls, aged 20–59 years, were recruited from their relatives, hospital visitors, and hospital personnel, during the same period. Participants with following conditions were excluded: 1) pregnancy or lactating, 2) receiving cancer treatment, organ transplantation, ≥0.5 mg/kg/day of prednisolone or equivalent, or immunomodulating treatment, 3) impaired renal function (creatinine clearance <30 mL/min), and 4) impaired liver function as defined by Child-Pugh C.

All participants who had no protective antibody to at least one of the three viruses were recruited into the second phase of the study. In this phase, participants were vaccinated with a single dose of MMR vaccine (GlaxoSmithKline Biologicals) at deltoid region by WK. Each 0.5 ml of vaccine contained at least 1000 TCID50 of Schwarz measles strain, at least 1000 TCID50 of RIT 4385 mumps, and at least 1000 TCID50 of Wistar RA 27/3 rubella strains. Antibodies to MMR were measured at week 8 to12, and week 48 after vaccination.

### Sample size calculation

Based on a previous study in Thai adults aged ≥25 years, the prevalences of seroprotective antibody to measles, mumps, and rubella were 90.6–96.3 %, 86.2–94.9 %, and 85.6–96.9 %, respectively [6]. We estimated that the prevalence of protective antibody to each of these viruses was 80 % among HIV-infected adults and 94 % among HIV-uninfected controls. For the first phase of the study, a sample size of 103 per group was required to significantly detect differences between them, at the power of 80 % using a cutoff for a two-sided alpha level of 0.05. However, we over-enrolled participants in both groups in order to have adequate number of participants without protective antibody for the second phase of the study.

### Data collection

Demographic and clinical data were collected using a preprinted data collection form including age, sex, recall of prior measles, mumps, or rubella vaccination, anti-measles IgG antibody level, anti-mumps IgG antibody titer, and anti-rubella IgG antibody level at the time of enrolment. The levels of antibody to the three viruses were also measured at 8 to 12 weeks, and 48 weeks after vaccination in those who were enrolled into the second phase of the study. In addition, CD4 cell count and plasma HIV-1 RNA within 6 months before enrolment were collected for all HIV-infected adults. Local and systemic adverse effects of the vaccine were also collected.

### Measurement of antibody levels to measles, mumps, and rubella

Laboratory tests were performed at the National Institute of Health, Department of Medical Sciences, Ministry of Public Health, Bangkok, Thailand. The indirect ELISA using Enzygnost® Anti-Measles Virus/IgG, Anti-Parotitis-Virus/IgG, Anti-Rubella-Virus-IgG (Siemens, Marburg, Germany) were performed following the manufacturer’s instructions. The optical density (OD) readings were interpreted as negative, equivocal, and positive if the delta OD was <0.1 (cut off), 0.1–0.2, and >0.2, respectively. Positive delta ODs were then converted to international units, as described in the package insert [17–19]. Protective antibody levels were defined as an antibody level ≥320 mIU/mL, antibody titer ≥1:500, and antibody level  ≥10 IU/mL for measles, [10] mumps, [20] and rubella, respectively [21].

### Statistical analysis

Data were presented in mean ± SD, median and IQR, number (%) as appropriate. Continuous variables were compared using Student’s *t*-test or Mann Whitney-*U* test, and categorical variables were compared using Chi-2 test and Fisher’s exact test. Statistical significance was set as 2-tailed, with *p*-value of <0.05. The Bonferroni correction was used for multiple comparisons. All statistical analyses were performed using Stata statistical software version 11.0 (Stata Statistical Software: Release 11.0, Stata Corporation, College Station, TX, 2009).

The study was approved by the Faculty of Medicine, Chiang Mai University Ethical Committee. The trial was registered to the ClinicalTrials.gov (NCT02724852) on March 31, 2016.

## Results

### Seroprevalence of antibodies to measles, mumps, and rubella

Five hundred HIV-infected adults and 132 HIV-uninfected controls were enrolled. Among HIV-infected adults, 218 (43.6 %) were male; the median age was 41 years (IQR 36, 48), and the median CD4 cell count was 470 cells/mm^3^ (IQR 364, 594). Among HIV-uninfected adults, 35 were male (26.5 %), and the median age was 38 years (IQR 30.5, 48). Characteristics of HIV-infected adults and HIV-uninfected controls are shown in Table 1.

**Table 1:** Characteristics of HIV-infected adults and HIV-uninfected controls

Characteristics	HIV-infected adults (*N* = 500)	HIV-uninfected controls (*N* = 132)	*p*-values
Male	218 (43.5)	35 (26.5)	<0.001
Age (years)	41 (36, 48)	38 (30.5, 59)	0.001
History of measles or mumps or rubella vaccination			<0.001
Yes	60 (12.0)	12 (9.1)	
No	231 (46.2)	9 (6.8)	
Uncertain	209 (41.8)	111 (84.1)	
History of mumps			0.161
Yes	39 (7.8)	6 (4.6)	
No	454 (90.8)	126 (95.5)	
Uncertain	7 (1.4)	0 (0)	
History of measles			0.825
Yes	12 (2.4)	2 (1.5)	
No	481 (96.2)	128 (97.0)	
Uncertain	7 (1.4)	2 (1.5)	
History of rubella			0.039
Yes	2 (0.4)	3 (2.3)	
No	491 (98.2)	129 (97.7)	
Uncertain	7 (1.4)	0 (0)	

The prevalences of protective antibodies to measles, mumps, and rubella and the antibody levels across age groups are shown in Tables 2 and 3, respectively. Among HIV-infected adults, the prevalence of protective antibody to measles was significantly lower among adults aged 20–29 years than other age groups (*p*-values < 0.05). There were no differences of the prevalence of protective antibody to mumps across age groups. The prevalence of protective antibody to rubella was lower among adults aged 40–49 years than aged 30–39 (*p*-value = 0.016), and 50–59 years (*p*-value = 0.035). However, overall, there were no differences in prevalences of protective antibodies to all three viruses across all age groups. Among HIV-uninfected controls, there were also no differences in prevalences of protective antibodies to all three viruses across all age groups. However, we observed a higher anti-mumps IgG titer among those aged 50–59 years than all other age groups (*p*-values < 0.05).

**Table 2:** Prevalences of protective antibodies against measles, mumps, and rubella in HIV-infected adults and HIV-uninfected controls across age groups

Age group (years)	Measles (number, %)			Mumps (number, %)			Rubella (number, %)		
	HIV-infected adults	HIV-uninfected controls	*p*-value	HIV-infected adults	HIV-uninfected controls	*p*-value	HIV-infected adults	HIV-uninfected controls	*p*-value
20–29	19/24 (79.2)	28/30 (93.3)	0.221	15/24 (62.5)	20/30 (66.7)	0.781	23/24 (95.8)	29/30 (96.7)	1.000
30–39	186/192 (96.9)	42/42 (100)	0.595	101/192 (52.6)	29/42 (69.1)	0.060	168/192 (87.5)	36/42 (85.7)	0.799
40–49	170/182 (93.4)	29/30 (96.7)	0.699	95/182 (52.2)	17/30 (56.7)	0.697	142/182 (78.0)	25/30 (83.3)	0.634
50–59	96/102 (94.1)	30/30 (100)	0.336	64/102 (62.8)	23/30 (76.7)	0.192	90/102 (88.2)	28/30 (93.3)	0.736
Overall	471/500 (94.2)	129/132 (97.7)	0.120	275/500 (55.0)	89/132 (67.5)	0.010	423/500 (84.6)	118/132 (89.4)	0.209

**Table 3:** IgG antibody levels to measles, antibody titers to mumps, and antibody levels to rubella in HIV-infected adults and HIV-uninfected controls across age groups

Age group (years)	Anti-measles IgG level (mIU/mL)			Anti-mumps IgG titer (titer 1:xxxx)			Anti-rubella IgG level (IU/mL)		
	HIV-infected adults	HIV-uninfected controls	*p*-value	HIV-infected adults	HIV-uninfected controls	*p*-value	HIV-infected adults	HIV-uninfected controls	*p-*value
20–29	1743 (1101, 6816)	4383.5 (1526, 6195)	0.399	919 (202, 2009.5)	976 (414, 1588)	0.803	44.5 (18.5, 86)	44.5 (21, 73)	0.741
30–39	3327 (1636, 5378)	5015 (2724, 6652)	0.007	605 (220, 1546)	967 (403, 1664)	0.064	43.5 (18, 105)	28 (14, 60)	0.071
40–49	2949 (1416, 6010)	5023.5 (2990, 7735)	0.345	552 (138.5, 1522)	720.5 (174, 1558)	0.777	41 (12, 106)	52 (12, 70)	0.976
50–59	3115.5 (1568, 5737)	5938 (2591, 6986)	0.066	905.5 (315.5, 2199.5)	1273.5 (533, 2716)	0.074	54 (17, 101.5)	59.5 (13, 103)	0.879
Overall	3055 (1491, 5737)	4994 (2531.5, 6718.5)	<0.001	644 (190, 1777)	976 (313, 2057)	0.010	44 (16, 105)	38 (13, 89)	0.305

Although the prevalence of protective antibody to mumps was lower in HIV-infected adults than in HIV-uninfected controls (*p*-value = 0.010), there were no differences in the prevalences of protective antibodies to all three viruses between HIV-infected adults and HIV-uninfected controls in all age groups. The antibody levels against measles were significantly higher in HIV-uninfected controls than HIV-infected adults in those aged 30–39 years. The antibody titers against mumps and the antibody levels against rubella were not different between HIV-infected and HIV-uninfected controls in all age groups.

### Serologic responses after vaccination

**Fig. 1:**
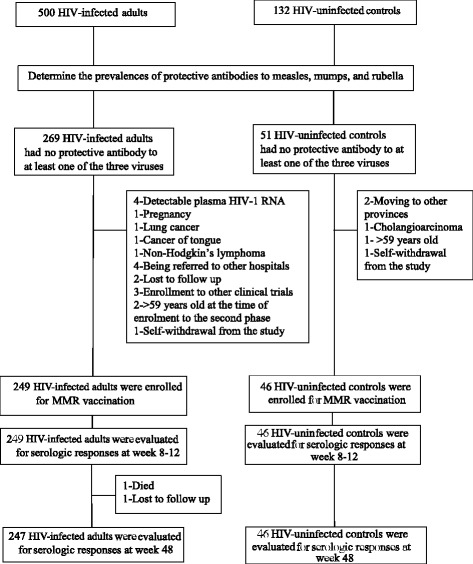
Flow of participants

There were 269 HIV-infected adults and 51 HIV-uninfected controls who had no protective antibody to at least one of the three viruses. Two-hundred and forty-nine HIV-infected adults and 46 HIV-uninfected controls were recruited into the second phase of the study. (Fig. 1) The reasons for not participating in the second phase of the study among 20 HIV-infected adults included 1) detectable plasma HIV-1 RNA (4 participants), 2) pregnancy (1), 3) diagnoses of lung cancer (1), cancer of tongue (1), and non-Hodgkin’s lymphoma (1), 4) being referred to other hospitals (4), 5) lost to follow up (2), 6) enrolment to other clinical trials (3), 7) >59 years old at the time of enrolment to the second phase (2), and 8) self-withdrawal from the study (1). The reasons in those five HIV-uninfected controls included 1) moving to other provinces (2), 2) diagnosis of cholangioarcinoma (1), 3) >59 years old (1), and 4) self-withdrawal from the study (1). Two HIV-infected adults were not tested for serologic responses at the 48^th^ week; one patient died at home 32 weeks after vaccination and the other was lost to follow up.

One hundred and six of 249 HIV-infected adults and 13 of 46 HIV-uninfected controls were male (*p*-value 0.074). The median age of HIV-infected adults was 41 years (IQR 35, 46) and that of HIV-uninfected controls was 38 years (IQR 31, 45) (*p*-value = 0.044). The median CD4 cell count among HIV-infected adults was 455 cells/mm^3^ (IQR 355, 589). The prevalences of protective antibodies against measles, mumps, and rubella at baseline and after vaccination in HIV-infected adults and HIV-uninfected controls are shown in Table 4. Among HIV-infected adults, 11, 83, and 30 % had no protective antibodies against measles, mumps, and rubella, respectively. For HIV-uninfected controls, 5, 65 and 24 % had no protective antibodies against measles, mumps, and rubella, respectively.

**Table 4:** Prevalences of protective antibodies to measles, mumps, and rubella after vaccination in HIV-infected adults and HIV-uninfected controls

	HIV-infected adults			HIV-uninfected controls		
	Baseline	Week 8–12	Week 48	Baseline	Week 8–12	Week 48
Measles						
Presence of protective antibody to measles						
All	222/249 (89.2)	240/249 (96.4)	234/247 (94.7)	44/46 (95.7)	46/46 (100)	44/46 (95.7)
Detectable baseline protective antibody to measles	-	220/222 (99.1)	219/220 (99.6)	-	44/44 (100)	43/44 (97.7)
No baseline protective antibody to measles	-	20/27 (74.1)	15/27 (55.6)	-	2/2 (100)	1/2 (50)
Anti-measles IgG level (mIU/mL)						
All	2654 (1132, 4768)	2771 (1315, 4911)	2604 (1208, 5061)	3353.5 (2035, 5834)	3221.5 (1367, 5232)	2863.5 (1659, 5164)
Detectable baseline protective antibody to measles	2946 (1560, 5082)	3022.5 (1674, 5225)	2993 (1572.5, 5291)	3450.5 (2215, 5970.5)	3254.5 (1427.5, 5313.5)	2977 (1720.5, 5508.5)
No baseline protective antibody to measles	200 (148, 269)	616 (289, 2049)	394 (228, 961)	137 (129, 145)	1791.5 (342, 3241)	965.5 (226, 1705)
Mumps						
Presence of protective antibody to mumps						
All	42/249 (16.9)^*^	176/249 (70.7)^**^	182/247 (73.7)^***^	16/46 (34.8)^*^	40/46 (87.0)^**^	40/46 (87.0)^***^
Detectable baseline protective antibody to mumps	-	40/42 (95.2)	42/42 (100)	-	13/16 (81.3)	14/16 (87.5)
No baseline protective antibody to mumps	-	136/207 (65.7)^****^	140/205 (68.3)^*****^	-	27/30 (90)^****^	26/30 (86.7)^*****^
Anti-mumps IgG titer (1:xxxx)						
All	220 (61, 431)	1086 (423, 2818)	886 (457, 1820)	247 (107, 399)	1821 (1223, 3375)	1191.5 (696, 1997)
Detectable baseline protective antibody to mumps	1221 (785, 2765)	2550 (1198, 4523)	1836.5 (1089, 3443)	474 (148, 890)	2280 (1278, 4006)	1653 (804, 2054.5)
No baseline protective antibody to mumps	151 (36, 323)	923 (337, 2227)	777 (361, 1391)	200 (101, 328)	1695 (1223, 2819)	1094.5 (629, 1997)
Rubella						
Presence of protective antibody to rubella						
All	174/249 (69.9)	244/249 (98.0)	241/247 (97.6)	35/46 (76.1)	46/46 (100)	46/46 (100)
Detectable baseline protective antibody to rubella	-	174/174 (100)	171/172 (99.4)	-	35/35 (100)	35/35 (100)
No baseline protective antibody to rubella	-	70/75 (93.3)	70/75 (93.3)	-	11/11 (100)	11/11 (100)
Anti-rubella IgG level (IU/mL)						
All	27 (5, 74)	51 (27, 90)	47 (22, 87)	15 (7, 40)	47.5 (26, 86)	37 (21, 61)
Detectable baseline protective antibody to rubella	47.5 (23, 99)	57.5 (27, 103)	42.5 (22, 91.5)	35 (21, 55)	47 (26, 86)	35 (21, 55)
No baseline protective antibody to rubella	1 (1, 1)	45 (25, 68)	52 (23, 79)	1 (1, 7)	51 (11, 95)	42 (10, 42)

^*^ *p*-value = 0.005, ^**^ *p*-value = 0.022, ^***^ *p*-value = 0.061, ^****^ *p*-value = 0.007, ^*****^ *p*-value = 0.039

After vaccination, overall, there were no differences between HIV-infected adults and HIV-uninfected controls in the seroprotective rates against all three viruses. For mumps, the seroprotective rates were lower among HIV-infected adults at baseline (*p*-value = 0.005), at 8–12 weeks (*p*-value = 0.022), and at 48 weeks after vaccination (*p*-value = 0.061). For those who had no protective antibody at baseline, the seroprotective rates was higher among HIV-uninfected controls than HIV-infected adults at 8–12 weeks (*p*-value = 0.007) and 48 weeks after vaccination (*p*-value = 0.039).

### Adverse effects of vaccination

Adverse effects during the first 72 h after vaccination included pain at the injection site (ten participants, 3.4 %), fatigue (8, 2.7 %), headache (7, 2.4 %), fever (6, 2.0 %), and local reactions at the injection site (3, 1.0 %). All these adverse effects resolved spontaneously without treatment.

## Discussion

This study demonstrated that in northern Thailand, the prevalence of seroprotective antibody to mumps in healthy adults was significantly lower than those to measles and rubella. These findings are similar to those in the study among healthy adults from northern Greece where the prevalence of seroprotective antibody to mumps was lower than that to measles [22]. However, our findings are different from those in the study among Thai adults in which the prevalences of seroprotective antibodies to all three viruses were comparably high [6]. Among HIV-infected adults, we found that the prevalence of seroprotective antibody as well as the level of antibody to mumps were significantly lower than those to measles and rubella. These findings are similar to those in studies among HIV-infected children in Thailand, [15] HIV-infected adults in England [23] and Austria,[24] but in contrast with findings in the study from the US where the prevalence of seroprotective antibody to measles was the lowest [14]. The differences among studies might be explained by the difference in the national vaccination program and also the prevalence of natural infection in each community. In Thailand, as previously described, measles vaccine and rubella vaccine were incorporated to the national vaccination program in 1984 and 1993, respectively, while MMR vaccine replaced vaccines for single virus in 1997. Therefore, the prevalence of seroprotective antibody to mumps would be lower than those for measles and rubella. Interestingly, a study among Thai population in 2004 revealed that the prevalence of seroprotective antibody to mumps was more than 80 %, and comparable to those to measles and rubella; this high prevalence of antibody to mumps corresponded with the transmission of mumps virus during that period [6, 12]. 

We found that there were no differences in the prevalences of seroprotective antibodies to all three viruses across all age groups for both HIV-infected adults and HIV-uninfected controls. In addition, there was no difference in the prevalences of seroprotective antibodies to all three viruses between HIV-infected adults and HIV-uninfected controls in each age group. However, this must be interpreted with caution from the small number of participants in each age group.

In the second phase of our study to determine the serological responses to MMR vaccination in participants who had no protective antibody to any of the three viruses, we found that the prevalence of seroprotective antibody to measles at baseline were very high in both HIV-infected adults and HIV-uninfected controls. Therefore, minority of them required measles vaccination. Among HIV-infected adults, the seroprotective rate against measles and mumps at 8 to 12 weeks after MMR vaccination was lower among those who had no protective antibody at baseline compared to those who had protective antibody at baseline (*p*-value < 0.001). The immunity to measles and mumps persisted through week 48 after vaccination for those who had protective antibody at baseline but not for those who had no protective antibody at baseline. These findings are similar to the studies among HIV-infected Thai children and HIV-infected adults in Mexico [15, 16]. However, we found that the seroprotective rate against rubella after MMR vaccination was almost 100 % and the immunity to rubella persisted through week 48. The excellent antibody response to the rubella component of MMR vaccine has been shown in several studies among children [11, 15]. Our study found that most of the adverse reactions from MMR vaccine were mild and self-limited. None experienced serious adverse events.

Our study had some limitations. This study was not a pair-matched case–control study; therefore, there were some differences in baseline demographic characteristics between groups. Compared to the HIV-infected adults, the HIV-uninfected controls in our study were younger and had more female; however, there were no significant differences in the prevalences of seroprotective antibodies at baseline as well as seroprotective rates to all three viruses after MMR vaccination among different age groups and genders (data not shown). In addition, we also found the imbalance in history of measles, mumps, or rubella vaccination, and a recalled history of infections due to those viruses between groups. However, this imbalance had no effect to the results since there were no correlations between history of vaccination or recalled history of infections due to those viruses with the prevalences of seroprotective antibodies at baseline as well as seroprotective rates to those viruses after MMR vaccination (data not shown).

## Conclusions

This study demonstrated that in northern Thailand, the majority of HIV-infected adults had protective antibody to measles which is the most contagious virus among the three virus components of MMR vaccine and can cause serious complications. Lack of immunity against mumps was more prevalent in our study. Seroprotective rates against measles and mumps after MMR vaccination were lower and the immunities persisted shorter in those who had no protective antibody at baseline. Since the MMR vaccine is safe and the history of prior vaccination or infections are unreliable, MMR vaccination should be considered in all HIV-infected adults receiving antiretroviral therapy who achieve undetectable plasma HIV-1 RNA and CD4 cell count ≥200 cells/mm^3^.

### Ethics approval and consent to participate

The study was approved by the Faculty of Medicine, Chiang Mai University Ethical Committee. Written informed consent was obtained before enrolment.

### Consent for publication

Not applicable.

### Availability of data and materials

Data will not be shared as the local IRB has no policy to share the data without prior permission.

## Abbreviations

cART: combination antiretroviral therapy
HIV: human immunodeficiency virus
MMR: measles, mumps, and rubella
